# An Investigation on the Effect of Extremely Low Frequency Pulsed Electromagnetic Fields on Human Electrocardiograms (ECGs)

**DOI:** 10.3390/ijerph13111171

**Published:** 2016-11-23

**Authors:** Qiang Fang, Seedahmed S. Mahmoud, Jiayong Yan, Hui Li

**Affiliations:** 1 School of Engineering, RMIT University, Melbourne 3000, Victoria, Australia; Seedahmed.Mahmoud@rmit.edu.au; 2 Department of Biomedical Engineering, Shanghai University of Medicine and Health Sciences, Shanghai 201318, China; yanjy@sumhs.edu.cn; 3 The Fifth Affiliated Hospital of Southern Medical University, Guangzhou 510900, China; ny5y@foxmail.com

**Keywords:** electrocardiograms (ECGs), extremely low frequency pulse electromagnetic fields (ELF-PEMF), time frequency analysis

## Abstract

For this investigation, we studied the effects of extremely low frequency pulse electromagnetic fields (ELF-PEMF) on the human cardiac signal. Electrocardiograms (ECGs) of 22 healthy volunteers before and after a short duration of ELF-PEMF exposure were recorded. The experiment was conducted under single-blind conditions. The root mean square (RMS) value of the recorded data was considered as comparison criteria. We also measured and analysed four important ECG time intervals before and after ELF-PEMF exposure. Results revealed that the RMS value of the ECG recordings from 18 participants (81.8% of the total participants) increased with a mean value of 3.72%. The increase in ECG voltage levels was then verified by a second experimental protocol with a control exposure. In addition to this, we used hyperbolic T-distributions (HTD) in the analysis of ECG signals to verify the change in the RR interval. It was found that there were small shifts in the frequency-domain signal before and after EMF exposure. This shift has an influence on all frequency components of the ECG signals, as all spectrums were shifted. It is shown from this investigation that a short time exposure to ELF-PEMF can affect the properties of ECG signals. Further study is needed to consolidate this finding and discover more on the biological effects of ELF-PEMF on human physiological processes.

## 1. Introduction

Over the past few decades, the beneficial therapeutic effects of selected non-ionising low-energy, time-varying electromagnetic fields (EMF) have been studied. This has led to an increased frequency of treatments for therapeutically resistant problems of the musculoskeletal system [1]. Moreover, a number of studies have investigated the therapeutic effects of extremely low frequency pulse electromagnetic fields (ELF-PEMF) in the following applications: relieving insomnia [2], accelerated bone repair and pain reduction [3,4], and dental sensory and cutaneous pain [5]. Studies [6,7] have also demonstrated that ELF-PEMF radiation facilitates the process of wound repair. A recent study [8] investigated the effects of ELF-PEMF radiation on the growth of bacterium *Staphylococcus aureus*, which plays a vital role in infecting wounded tissues. The results revealed that all irradiated *S. aureus* bacteria showed a decrease in their growth rate compared to the control samples. Graham et al. studied the relationship between field strength and biological response [9]. This study reported that a significant slowing of the heart rate occurred in the group exposed to the 9 kV/m, 20 μT combined field. The double-blind study was performed with magnetic or electric fields and reported that, for up to 5 μT or 20 kV/m, there are minimal or no effects on either the electroencephalogram (EEG) (evoked potentials) or the cognitive responses of human participants [9,10,11]. Moreover, studies in [12,13,14] reported that there is a weak association between exposure to ELF-EMF and leukemia and brain tumours.

Several studies have reported on the influence of ELF-PEMF exposure on electrocardiogram (ECG) signals. Examples from these studies include the ELF-PEMF effect on heart rate variability (HRV) alterations (the low frequency (LF) and high frequency (HF) ratio) [15,16,17], the effect on the 16 Hz/28.3 μT MF exposure to HRV [18], the effect on an intermittent 16.7 Hz MF exposure to heart rate (HR) [19], and the effect on time interval parameters derived from the ECG wave, such as the duration of P and QRS waves, the duration of PR and QT intervals, and corrected QT (QTc) [20]. Graham et al. studied specific exposure circumstances under which changes in heart rate variability (HRV) will occur [21]. In that study, (1) during exposure to a much higher intensity field (resultant flux density = 127.3 μT), the cardiac autonomic control was measured; (2) across three relevant test conditions (intermittent and continuous field exposure, during a no-exposure, and a control condition), HRV values measured from the same individual were compared; (3) whether the precise timing of when the magnetic field switched on or off in relation to the cardiac cycle results in the phase-resetting of human cardiac rhythm ECG data was determined. In another study, Graham et al. performed digital Fourier transform analysis for ECG signals [21]. However, this technique does not reveal the multi-component nature of the ECG signal [22]. Their studies concluded that exposure to strong ELF magnetic fields does not alter cardiac autonomic control mechanisms [21]. In one study [23], it was reported that the radiofrequency of cell phones prolonged the QT interval in humans with or without ischemic heart disease and interferes with the voltage property of ECG records in male patients with myocardial ischemia. Results in [24] revealed that long-term exposure to cell phone EMF increases the liability for hypertension reflected on the ECG and cardiac weights, accompanied by histological changes in the myocardium. One investigation found no indication of an association between occupational extremely low-frequency magnetic fields (ELF-EMF) exposure and risk of cardiovascular disease mortality [25].

Thus, a number of studies have reported the beneficial therapeutic effects of ELF-EMF; however, few studies have investigated its effect on ECG signal morphology and time intervals. Therefore, in this study, we investigate the possible biological effects of ELF-PEMF on electrocardiograms (ECGs). The aim of this study was to investigate whether there are any alterations in the ECG due to ELF-PEMF exposure. Initially, the root mean square (RMS) values for the ECG signal were measured and compared. Additionally, we measured and compared some of the ECG intervals (PR, RT, QT, and RR) before and after exposure to the EMF [26]. Then, we use the time-frequency approach with the optimal smoothing kernels (HTD) in the analysis of ECG signals to verify the change in ECG time intervals [27]. The control group followed the same experiment procedures with a sham exposure to investigate whether the change observed is due to the exposure or due to rest over time.

## 2. Materials and Methods

### 2.1. Participants

Twenty-two healthy volunteers (aged 20–38, 16 males and 6 females) were recruited to participate in this study. All participants were right-handed and healthy, without any medical or psychiatric disorders. They were not taking any medication that could affect mental or neural activities. An additional 11 volunteers participated in a control exposure in which there was no ELF-PEMF applied to the participants, to verify the validity of the experimental protocol considered in this investigation. The experimental protocol was approved by the Ethics Committee of RMIT University (Project ID: RMIT-RIF-FANGMAHMOUD). All of the participants gave written informed consent for the experiment.

### 2.2. Source of ELF-PEMF Exposure

**Figure 1:**
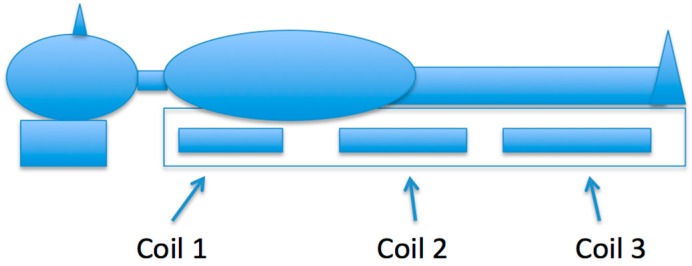
Exposure setup. The subject lies on their back on the mat with chest over Coil 1 (2.33 μT), waist over Coil 2 (5.235 μT), and legs over Coil 3 (6.45 μT).

The ELF-PEMF generation system used in this investigation was designed and is manufactured by MEDEC Ltd., Salter Point, Australia [28]. It has a Class II certificate (CQ041744-II) with the European Medical Devices Directive 93/42/EEC. The ELF-PEMF generation system, which is a 1.5 m × 0.9 m rectangle sized foam mat with a plastic cover, has three pairs of different sized coils embedded inside (See Figure 1). The system has the flexibility to select different frequencies and intensities. Our research chose the ELF-PEMF fields setting for the top, middle, and bottom coils of the mat to be 2.33 μT, 5.235 μT, and 6.45 μT, respectively, with a 16 Hz operating frequency. In this setting, the effect of the electromagnetic fields for the top, middle, and bottom coils decreased at 15 cm, 30 cm, and 32 cm, respectively. These distances were measured from the centre of each coil above the mat where the subject lay. The subject lies on his or her back with their body on the mat and their head just off the mat. The chest is over the coil with low intensity (2.33 μT), the waist lies over the coil with medium intensity (5.235 μT), while the legs lie over the coil with higher intensity (6.45 μT). The exposure setting is illustrated in Figure 1. In this study, the magnetic flux density for the three coils was verified by direct measurement using an EFA-200 EMF analyser and external B-field probe with a diameter of 3 cm and a measurement accuracy of 6%. The ELF-PEMF generation system creates PEMF with four identical saw-tooth-like signal (0–100 Hz) bundles and delays, each lasting 20 ms. These saw-tooth-like controlled signals oscillate between 0.4 V and −0.8 V. 

### 2.3. ECG Equipment

ECG is a vital human physiological signal that can be used to diagnose the condition of the heart. The ECG time intervals, such as PR, RT, QT, and RR, are the main propagation characteristics that relate directly to phases of the cardiac electric conduction. The P-wave is caused by atrial depolarisation, and its typical duration is normally less than 0.12 s. The PR interval is the portion of ECG wave from the start of P-wave (onset of atrial depolarisation) to the beginning of QRS-complex (onset of ventricular depolarisation). Its duration is normally 0.12 to 0.20 s. The RR interval is the duration between two adjacent R waves, which is the reciprocal of the heart rate. The prominent QRS complex is caused by the ventricle depolarisation and has a typical duration of 0.06 to 0.10 s. Within the period of the ELF range, those ECG time intervals have high diagnostic values for various cardiovascular diseases. 

Lead-I ECG electrodes were connected to the BIOPAC systems (ECG Module). The ECG module consists of an instrumentation amplifier (IA) and a 50 Hz notch filter. The IA has a very high unwanted signal rejection capability and a variable gain (the gain was set to 1000). The sampling rate for ECG was set to 1000 samples/s. Lead-I ECG is the voltage between the left arm (LA) electrode (positive) and right arm (RA) electrode and is one of the three bipolar leads. Lead-I ECG provides a left lateral view of the heart. Though Leads I, II, and III are often used simultaneously to monitor multiple regions of the heart, we chose Lead I for this study because it is most often used for cardiac monitoring. Moreover, we are interested in comparing the time intervals of ECG. Leads I, II, and III will provide the same information in this regard. The ECG data was recorded with AcqKnowledge software (v.3.7.1, BIOPAC Systems, Inc., Goleta, CA, USA) in ASCII text files and processed by Matlab. Four ECG time intervals, namely PR, RT, QT, and RR, were chosen for this study to further investigate whether the ECG signals could be altered when exposed to ELF-PEMF. 

### 2.4. Recording Protocols

For this study, we considered two recording protocols. In the first protocol (protocol-1), the participant was asked to relax and lie on their back for five minutes. Then, a 10 min duration of ELF-PEMF is applied to the participant, after which a 30 s recording of ECG signal was taken. The data recording took place in a dim room at 20 °C, inside a Faraday cage (1.95 × 1.83 × 2.67 m) to shield the subjects from the 50 Hz electric field. Experiments were conducted on weekdays (excluding weekends) between 10 a.m. and 4 p.m. Lead-I was considered in the ECG recording. Measurements of 30 s durations for the ECG signal were taken immediately after the exposure. The experiment was conducted under single-blind conditions. The second protocol (protocol-2) was carried out to verify the validity of the first protocol (protocol-1). In the second protocol, only ECG was measured, and there was no ELF-PEMF exposure.

There are a number of limitations when considering a single-blind method over a double-blind method. However, precaution measures were considered to reduce the limitation of the single-blind method: (1) participants were randomised for the experiment group and control group; (2) the analysis procedures and related software were prepared beforehand; and (3) the experiment was carried out with 3 researchers present, where no hint was given to the participants as to whether they were subject to the experiment or a sham. 

## 3. Experiment and Results

The RMS values of the ECG subjects’ data were calculated while the selected time intervals of ECG were measured. The RMS value is most commonly used in measuring the amplitude of biosignals. The amplitude of a biosignal expresses the magnitude of the energy or power of that signal. Measurement of RMS in different conditions affecting a biological system can give an index of the changes related to that particular effect. The significance of the RMS changes of ECG will be calculated using a Student’s *t*-test. The RMS for a discrete function *y(n)* is given by

$$Y_{rms}=\;\sqrt{\frac{1}{N}\overset{N}{\underset{i=1}{\sum }}y_{i}^{2}}$$

where *N* is the length of the data *y*. ECG results will be presented in the following subsections.

### 3.1. ECG Results

Thirty seconds of artefact-free Lead-I ECG data were recorded from each participant (total 22 participants) under protocol-1 and protocol-2, respectively. The RMS values, time interval analysis, and the time-frequency analysis techniques were considered for the set of ECG data of protocol-1. The RMS analysis was used as well for the set of data recorded with protocol-2 to validate the finding in protocol-1.

### 3.2. RMS and Time Intervals Analysis

The RMS values of the raw data for all participants were evaluated using Matlab. Moreover, the time intervals of PR, RT, QT, and RR before and after exposure were measured and compared for the data acquired from the experiment group. 

Table 1 shows the RMS values of the ECG signal for the 22 participants before and after the EMF exposure in the experiment group (protocol-1). The mean-RMS value of all participants of the ECG after exposure was increased by 0.0025 volts and the mean of the change rate is 3.72%. A paired *t*-test was conducted to statistically analyse whether there were any significant differences between the RMS before and after EMF exposure. The result of the *t*-test for the ECG is outlined in Table 2, where *p*-values are considered significant at less than 0.05.

From the analysis of the RMS values of the ECG for all participants, the results showed that there is a small increment in the ECG RMS level for most of the participants. Eighteen participants out of a total of 22 experienced increments in their RMS after EMF exposure. The *t*-test analysis (see Table 2) for the mean RMS level of the ECG indicates that there were statistically significant levels of difference in the ECG before and after ELF-EMF exposure. These results can be seen from Table 2 in the ECG Lead I where *p* = 0.0030 (*p* < 0.05). The 95% confidence interval of this significant result suggested that there was an increase in mean ECG potential after the exposure. The RMS analysis of the ECG data in protocol-2 was performed to verify whether the change was due to the extremely low frequency pulse electromagnetic fields (ELF-PEMF) exposure or due to the participant rest over time. Table 3 shows the RMS value of the ECG signal for the 11 participants after 5 min and 15 min of rest in the control group, respectively. From these results, it can be seen that the RMS values for all participants decreased over time. The mean RMS of all participants’ ECGs after 15 min of rest under the sham exposure was decreased by 0.0035 volts (−3.38%). In comparison between protocol-1 and protocol-2, the RMS values increased over time after the subjects were exposed to ELF-EMF (protocol-1), and decreased during the subjects’ rest time (protocol-2). 

**Figure 2:**
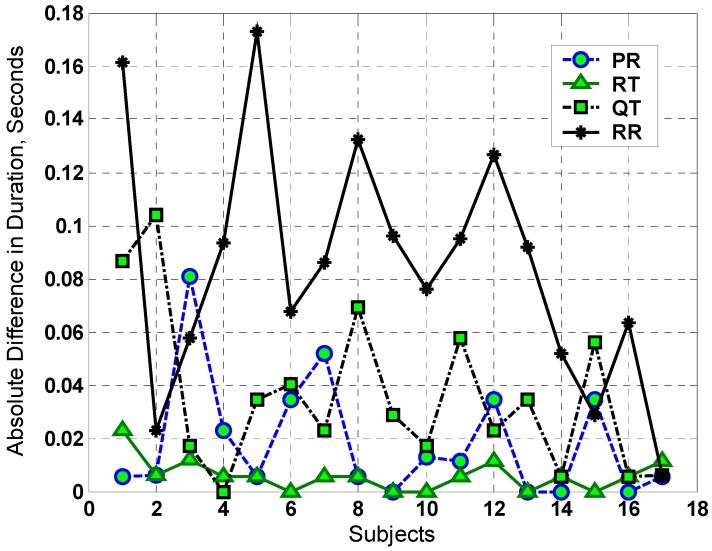
The absolute difference in duration for PR, RT, QT, and RR intervals of ECG before and after ELF-PEMF exposure.

The time intervals of the ECG signals were determined similar to the method in [26]. Figure 2 shows the absolute deviation for PR, RT, QT, and RR of ECG intervals before and after the exposure. From this figure, we observe that there are no significant changes in most of these intervals. The ELF-PEMF did not prolong the QT interval compared with the effect of mobile radio frequency on the QT interval [23]. This indicates that the ELF-PEMF did not significantly affect the electrical activity of cardiac tissue where these events originated. However, for the RR interval, there is a small change that may contribute to the variation in the heart rate (see Figure 2). To verify whether the variation in RR interval is due to the EMF exposure, time-frequency analysis was used for the analysis of the ECG signal, which is reported in the following section. 

### 3.3. Time-Frequency Analysis

All ECG signals were down-sampled to 50 samples/s for the time frequency analysis due to memory limitations. The mean of the ECG data was calculated and then subtracted from the ECG signal in order to remove any direct current (DC) offset. In addition to this, 15 s ECG signals were converted by Hilbert transform into their analytical forms and processed using the hyperbolic T-distributions (HTD) [27,29]. In all numerical simulations, we set the value of *α* = 0.05 for the HTD.

**Figure 3:**
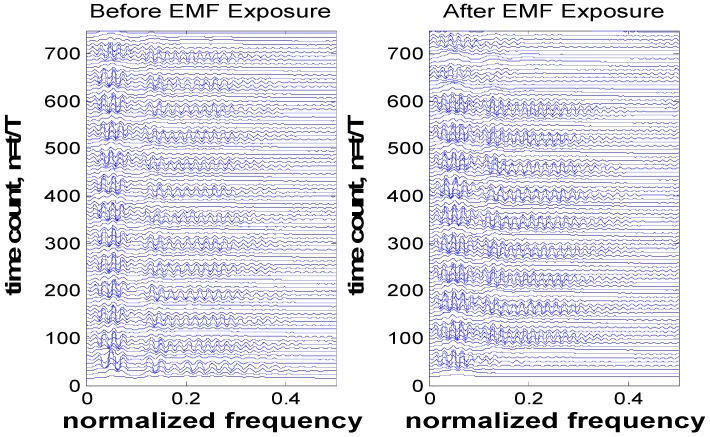
Hyperbolic T-distribution of the electrocardiogram (ECG) signal for participant-5 (with *α* = 0.05, sampling frequency = 50 samples/s, and signal length = 750 samples).

**Figure 4:**
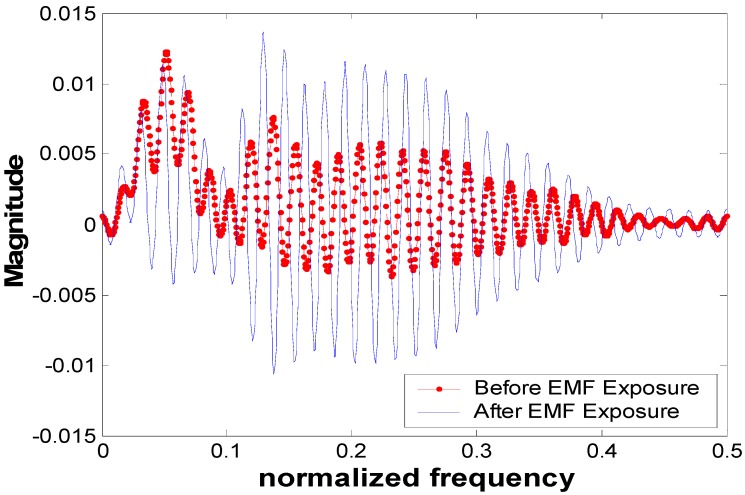
Frequency components of the ECG signal for participant-5 at the time instants *t* = 10 s.

Based on the observation of Figure 2, the time-frequency analysis for participant-5 (where this participant experienced larger variation in their RR interval relative to other participants) is shown in Figure 3. Other participants’ analysis results were similar (frequency shift before and after EMF exposure) but smaller in amplitude compared with this selected participant. Figure 4 shows the frequency components of the ECG signals for participant-5 at *t* = 10 s (such results can be seen at any time instant). From this figure, we observe that there is a small shift in the frequency-domain signal before and after EMF exposure. This shift has an influence on all frequency components of the ECG signals (where all spectrums have been shifted). This can be clearly observed and related to Figure 3 where the interval RR experienced small variation.

## 4. Discussion

The RMS values of the ECG signal after a short duration of ELF-PEMF exposure were noticeably increased. Eighteen participants, who represent 81.8% of the total participant population, experienced such an increment with an average increment of 3.72%. The 95% confidence interval of the significant result from the *t*-test analysis suggests that there was an increase in the mean of the ECG potential. In the control exposure group, the RMS values for all participants actually decreased over time due to the rest of the participants’ body. These results showed that the ELF-PEMF exposure noticeably changed the RMS values of the cardiac signals of the participants. The time interval analysis for the ECG showed that there was a small change in the RR interval (i.e., for subject-5, Figure 2, the RR interval changed by 0.18 s) before and after ELF-PEMF exposure. However, there were no significant changes for other intervals. The ELF-PEMF did not prolong the QT interval compared with the effect of mobile radio frequency on the QT interval [23]. This indicates that the ELF-PEMF did not significantly affect the electrical activity of cardiac tissue where these events originated. In the time-frequency analysis, the results showed that there was a small shift in the frequency-domain signal before and after EMF exposure. This shift has an influence on all frequency components of the ECG signals. This is due to the variation that occurred in the RR interval. In other words, the result from the time frequency analysis showed that the EMF exposure has an influence on RR interval. 

This investigation was a single-blind pilot study that has a potential for inherent defects. However, the high percentage of consistency (81.5%) and the high *t*-test confidence (95%) suggest a possible bio-effect of ELF-PEMF on ECG signals. Such a bio-effect could be due to capacitive coupling between the ELF-PEMF pulses and the relative narrow band ECG signal. This coupling could be due to significant frequency overlap between the ECG frequency components and the operating frequency range of the ELF-PEMF generation system (0–100 Hz). Nevertheless, it is still early to conclude that a short time exposure to ELF-PEMF and the coupling between the ELF-PEMF pulses and ECG components will affect the physiological processes of ECG.

## 5. Conclusions

The effects of extremely low frequency pulsed electromagnetic fields (ELF-PEMF) on human cardiac signals were investigated. In the ECG time interval analysis, the results showed a small change in RR interval. However, there were no significant changes for other intervals. This indicates that the only interval affected by EMF exposure on heart activity is the RR interval (the reciprocal of the heart rate). time-frequency analysis results show a small shift in frequency domain signal before and after ELF-PEMF exposure. This is due to the variation that occurred in the RR interval. Further study, especially a double-blind experiment, is needed to consolidate these findings and discover more on the effects of ELF-PEMF on human physiological processes. Conducting the ELF-PEMF exposure experiment over a number of days (excluding weekends) for at least one subject is another future research direction.

## Figures and Tables

**Table 1:** The root mean square (RMS) values of the electrocardiograms (ECG) and their change rates for the 22 participants (the RMS is in volts) using protocol-1.

Participant	Before the EMF Exposure	After the EMF Exposure	Change Rate
1	0.072	0.075	4.17%
2	0.060	0.063	5.00%
3	0.076	0.077	1.32%
4	0.098	0.094	−4.08%
5	0.051	0053	3.92%
6	0.063	0.065	3.17%
7	0.057	0.059	3.51%
8	0.068	0.067	−1.49%
9	0.080	0.081	1.25%
10	0.069	0.074	7.25%
11	0.068	0.073	7.35%
12	0.073	0.081	10.96%
13	0.069	0.071	2.90%
14	0.069	0.073	5.80%
15	0.074	0.082	10.81%
16	0.064	0.066	3.13%
17	0.063	0.065	3.17%
18	0.072	0.076	5.56%
19	0.081	0.079	−2.47%
20	0.073	0.072	−1.37%
21	0.062	0.065	4.84%
22	0.071	0.073	2.82%
Mean change rate			3.72%

EMF: Electromagnetic field.

**Table 2:** *t*-Test results for a comparison between exposure to extremely low frequency pulse electromagnetic field (ELF-EMF) and before exposure to ELF-EMF for the ECG signal.

Recording Site	ECG	
	95% CI (V)	*p*-value
ECG Lead I	−0.004, −0.001	0.0030

CI: confidence interval.

**Table 3:** The RMS values of the ECG and their change rates for the 11 participants (the RMS is in volts) using protocol-2.

Participant	After 5 Min of Rest	After 15 Min of Rest	Change Rate
1	0.105	0.098	−7.14%
2	0.075	0.072	−4.17%
3	0.073	0.070	−4.29%
4	0.069	0.067	−2.99%
5	0.116	0.112	−3.57%
6	0.073	0.071	−2.82%
7	0.152	0.148	−2.70%
8	0.162	0.158	−2.53%
9	0.158	0.155	−1.94%
10	0.112	0.110	−1.82%
11	0.160	0.155	−3.23%
Mean change rate			−3.38%
